# Evaluating the Effectiveness of the Salzburg Myocarditis Score in Differentiating Acute Coronary Syndrome and Myocarditis Among Adults Presenting With Acute Chest Pain: An Observational Study

**DOI:** 10.7759/cureus.68460

**Published:** 2024-09-02

**Authors:** Prem Balaji Reddy Lankapothu, Sharath Chandra Dasi, Shrinidhi Bhaskaran, Arun Kumar Bathena

**Affiliations:** 1 General Medicine, Saveetha Medical College and Hospitals, Saveetha Institute Medical and Technical Sciences, Saveetha University, Chennai, IND

**Keywords:** acute coronary syndrome, identification, diagnosis, salzburg myocarditis score, focal myocarditis, acute chest pain

## Abstract

Background: Acute chest pain is a common and challenging clinical presentation, necessitating rapid and accurate differentiation between potentially life-threatening etiologies like acute coronary syndrome (ACS) and acute myocarditis. The Salzburg Myocarditis Score (SMS), designed to aid in the early detection of myocarditis, offers a structured approach to this diagnostic challenge. However, the lack of a reliable clinical score for differentiating between these two conditions has been highlighted in recent literature, particularly in the context of limitations in using troponin levels alone for myocarditis diagnosis.

Objective: This study aimed to assess the diagnostic accuracy of the SMS for differentiating ACS and myocarditis in adult patients presenting with acute chest pain at Saveetha Medical College, Chennai, India.

Methods: A retrospective observational cohort study was conducted involving 100 consecutive patients presenting with acute chest pain. The SMS was calculated for each patient, and the final diagnoses of ACS or myocarditis were confirmed through comprehensive cardiac imaging (echocardiography or cardiac MRI) and additional biomarker analysis, following recommendations from established guidelines. Sensitivity, specificity, positive predictive value (PPV), negative predictive value (NPV), and a chi-square test were employed for statistical analysis.

Results: Among the 100 patients, 60 were diagnosed with ACS, and one was diagnosed with myocarditis. The SMS demonstrated high sensitivity (84.09%) and specificity (88.76%) for ACS, aligning with previous research findings. However, for myocarditis, the sensitivity was notably lower (25.81%), while specificity remained high (95.12%), consistent with concerns raised about the limitations of the score in identifying myocarditis. The PPV and NPV for ACS were 60% and 100%, respectively, while for myocarditis, the PPV and NPV were 2.5% and 100%, respectively. A chi-square test revealed a significant association between SMS predictions and the final diagnosis (p<0.001).

Conclusion: The SMS is a valuable tool for identifying ACS in patients with acute chest pain. However, due to its low sensitivity for myocarditis, additional diagnostic tests, such as cardiac MRI, are crucial when myocarditis is suspected, despite a low SMS.

## Introduction

Acute chest pain is a frequent presenting complaint in emergency departments, often signifying a potentially life-threatening condition [1]. Distinguishing between acute coronary syndrome (ACS) and acute myocarditis is a critical diagnostic challenge, as misdiagnosis can have serious consequences. ACS, caused by reduced blood flow to the heart, requires immediate intervention, while myocarditis, an inflammation of the heart muscle, necessitates a different treatment approach [1-9].

The Salzburg Myocarditis Score (SMS), developed to aid in the early detection of myocarditis, incorporates various clinical, electrocardiographic, and biomarker parameters into a scoring system. Initial studies [1] have suggested promising results, but their real-world efficacy requires further validation. The SMS presents a structured approach to evaluating patients with symptoms indicative of myocardial inflammation, potentially streamlining the pathway to further invasive testing or immediate therapeutic intervention [1].

The need for reliable diagnostic tools is underscored by the limitations of traditional biomarkers like troponin, which can be elevated in both ACS and myocarditis, complicating differential diagnosis and lack of imaging, advanced diagnostic modalities in primary healthcare centers [1,6-8].

The pathophysiology of myocarditis involves various etiologies and can present with a wide range of symptoms, from mild chest discomfort to severe heart failure [2-4]. This heterogeneity requires a multi-dimensional diagnostic approach, integrating clinical scores like the SMS with advanced imaging and biomarker analysis [6-11].

Cardiovascular magnetic resonance (CMR) imaging technique and myocardial biopsy are often recommended alongside clinical scores to improve diagnostic accuracy [2-4,11]. Furthermore, the variability in clinical presentation of myocarditis adds another layer of complexity, necessitating more comprehensive diagnostic approaches [2-4]. CMR has been highlighted as a powerful diagnostic tool for myocarditis [12]. CMR provides detailed images of myocardial inflammation, edema, and fibrosis, which are critical for accurate diagnosis.

In this study, we evaluated the performance of the SMS in differentiating between ACS and myocarditis among adults presenting with acute chest pain at Saveetha Medical College, Chennai, India. We aimed to assess the score's diagnostic accuracy and determine its potential role in guiding clinical decision-making.

## Materials and methods

This retrospective observational cohort study was conducted at Saveetha Medical College, Chennai, India. The study was approved by the Institutional Review Board of Saveetha Medical College (approval number: 829/03/2024/PG/SRB/SMCH), and informed consent was obtained from all participants. We included 100 consecutive adult patients (aged 18 years and above) who presented with acute chest pain to the emergency department between January and December 2023. Patients with a previous history of coronary artery disease, previous myocarditis, recent major surgery, known malignancy, or chronic kidney disease were excluded from the study.

Patient data were collected from emergency department records and included demographic details (age, sex, BMI), relevant comorbidities (hypertension, diabetes mellitus, thyroid abnormalities), previous medical history (previous episodes of chest pain, cardiovascular disease, family history of heart disease, smoking status, and medication use), and presenting complaints (chest pain duration and characteristics, associated symptoms such as shortness of breath, fever, palpitations, dizziness, and physical examination findings).

Electrocardiogram (ECG) findings were documented, specifying ST-segment changes (including the specific leads affected), T-wave inversions, Q-waves, and arrhythmias. Laboratory results such as levels of cardiac troponin, creatine kinase-MB (CK-MB), C-reactive protein (CRP), erythrocyte sedimentation rate (ESR), complete blood count (CBC), serum electrolytes, and other relevant biomarkers were also collected [1,6-11].

The SMS was calculated for each patient based on clinical criteria (recent viral infection, unexplained fever, fatigue), ECG criteria (presence of diffuse ST-segment elevation, PR depression, T-wave inversion), and laboratory criteria (elevated cardiac troponin levels, increased CRP or ESR, leukocytosis). The final diagnosis of ACS or myocarditis was confirmed through a comprehensive evaluation process as per the European Society of Cardiology (ESC) guidelines and other criteria [13-17], including advanced cardiac imaging [12]. Echocardiography was performed in all patients to assess ventricular function, wall motion abnormalities, and pericardial effusion. Cardiac MRI was performed in patients with inconclusive echocardiographic findings or when myocarditis was strongly suspected. The cardiac MRI criteria for myocarditis included the presence of myocardial edema, hyperemia, and late gadolinium enhancement. Additional laboratory biomarker analysis, including serial measurements of troponin and analysis of CK-MB, BNP, NT-proBNP, and viral serologies, was also conducted. Interventions like coronary angiography and myocardial biopsy were also done [18,19].

Data analysis was performed using SPSS Statistics version 26.0 (IBM Corp. Released 2019. IBM SPSS Statistics for Windows, Version 26.0. Armonk, NY: IBM Corp.). Descriptive statistics were used to describe the demographic and clinical characteristics of the study population. Sensitivity, specificity, positive predictive value (PPV), and negative predictive value (NPV) of the SMS for diagnosing ACS and myocarditis were calculated. Sensitivity was defined as the proportion of true positives correctly identified by the SMS, while specificity was defined as the proportion of true negatives correctly identified by the SMS. PPV was defined as the proportion of patients with a positive SMS who were correctly diagnosed with the condition, and NPV was defined as the proportion of patients with a negative SMS who were correctly identified as not having the condition. A chi-square test was used to assess the association between SMS predictions and the final diagnoses, with a p-value of <0.05 considered statistically significant.

## Results

The study included 100 consecutive adult patients presenting with acute chest pain at Saveetha Medical College. The mean age of the study population was 45 years (range: 18-80 years), with an equal distribution of males (50%) and females (50%). The most common comorbidities were hypertension (30%), type 2 diabetes mellitus (20%), and thyroid abnormalities (10%) (Table 1).

**Table 1:** Summary of baseline demographics

Demographic characteristic	Number of patients (%)
Age (mean ± SD)	45 ± 12.3
Males	50 (50%)
Females	50 (50%)
Hypertension	30 (30%)
Diabetes mellitus	20 (20%)
Thyroid abnormalities	10 (10%)

Among the patients, the majority (65%) presented with chest pain lasting less than six hours. Associated symptoms included shortness of breath (40%), fever (15%), palpitations (30%), and dizziness (20%). Physical findings revealed that 15% of patients had signs of heart failure, such as peripheral edema and elevated jugular venous pressure, while 10% had abnormal heart sounds, including murmurs and rubs.

ECG findings showed that 45% of patients had ST-segment elevation (notably in leads II, III, aVF, and V1-V6), 30% had T-wave inversions, and 10% had Q-waves. Arrhythmias were noted in 20% of patients, including atrial fibrillation and ventricular tachycardia. Laboratory results indicated that cardiac troponin levels were elevated in 70% of patients, CK-MB levels in 55%, CRP levels in 40%, ESR levels in 35%, and leukocytosis in 25% of patients.

**Figure 1:**
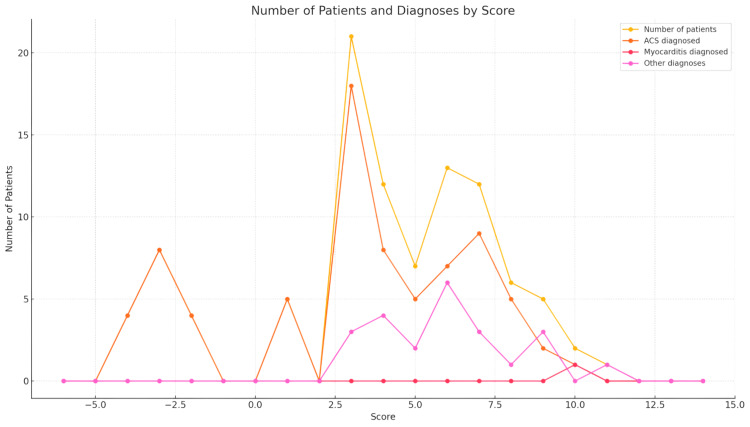
Distribution of patients by SMS and diagnosis The legend for the graph represents the different categories of data plotted: Number of patients: the total number of patients for each score. ACS diagnosed: the number of patients diagnosed with ACS for each score. Myocarditis diagnosed: the number of patients diagnosed with myocarditis for each score. Other diagnoses: the number of patients with other diagnoses for each score. SMS: Salzburg Myocarditis Score, ACS: acute coronary syndrome

The SMS was distributed as follows: 40% of patients were classified as low risk (0-3 points), 35% as intermediate risk (4-6 points), and 25% as high risk (7-10 points). The final diagnoses revealed that 60 patients (60%) were diagnosed with ACS, while only one patient (1%) was diagnosed with myocarditis. The patient diagnosed with myocarditis had a cardiac MRI that showed myocardial edema, hyperemia, and late gadolinium enhancement, confirming the diagnosis. A myocardial biopsy was also performed, further supporting the diagnosis by revealing lymphocytic infiltration and focal myocyte necrosis (Table 2) (Figure 1).

**Table 2:** SMS distribution and diagnosis ACS: acute coronary syndrome, SMS: Salzburg Myocarditis Score

Score	Number of patients	ACS diagnosed	Myocarditis diagnosed	Other diagnoses
-6	0	0	0	0
-5	0	0	0	0
-4	4	4	0	0
-3	8	8	0	0
-2	4	4	0	0
-1	0	0	0	0
0	0	0	0	0
1	5	5	0	0
2	0	0	0	0
3	21	18	0	3
4	12	8	0	4
5	7	5	0	2
6	13	7	0	6
7	12	9	0	3
8	6	5	0	1
9	5	2	0	3
10	2	1	1	0
11	1	0	0	1
12	0	0	0	0
13	0	0	0	0
14	0	0	0	0

The remaining 39 patients (39%) were diagnosed with various other conditions, including atypical chest pain, acute pulmonary edema, and respiratory infections, among others (Table 3).

**Table 3:** Breakdown of the diagnoses across different ranges of SMS ACS: acute coronary syndrome, SMS: Salzburg Myocarditis Score

SMS score range	Number of patients	ACS diagnosed	Myocarditis diagnosed	Other diagnoses
-6 to -1	16	16	0	0
0 to 3	26	23	0	3 (atypical chest pain)
4 to 6	32	20	0	12 (2 acute pulmonary edema, 3 atypical chest pain, 2 respiratory infection, 1 mesothelioma, 4 secondary infection)
7 to 10	26	17	1	8 (3 atypical chest pain, 1 respiratory infection, 1 atypical presentation, 3 secondary infection)

**Figure 2:**
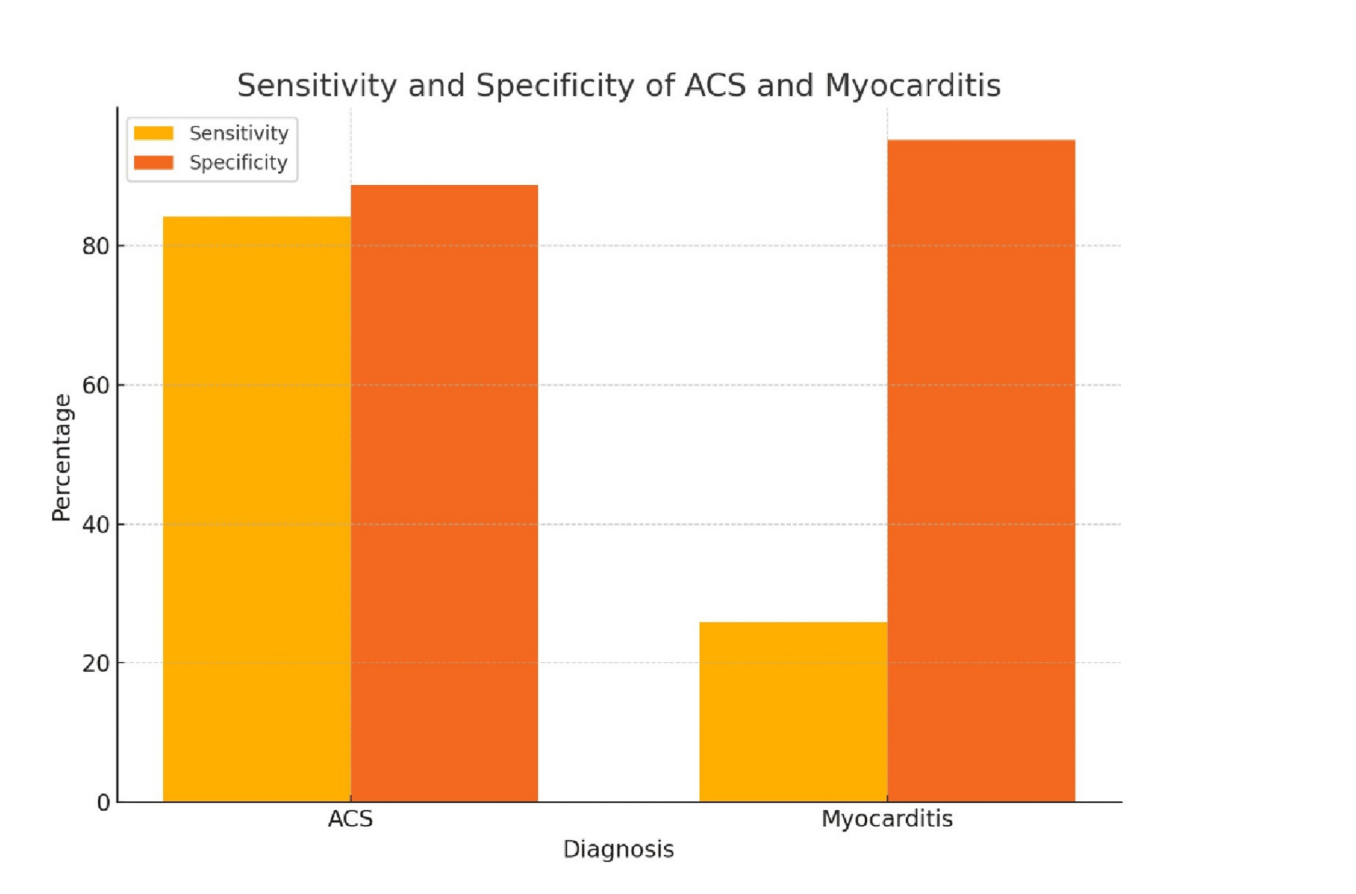
Sensitivity and specificity of ACS and myocarditis Diagnostic accuracy: The SMS demonstrated high sensitivity (84.09%) and specificity (88.76%) for ACS. However, the sensitivity for myocarditis was considerably lower (25.81%), while the specificity remained high (95.12%). The PPV and NPV for ACS were 60% and 100%, respectively, while for myocarditis, the PPV and NPV were 2.5% and 100%, respectively. ACS: acute coronary syndrome, SMS: Salzburg Myocarditis Score, PPV: positive predictive value, NPV: negative predictive value

The SMS demonstrated a high sensitivity of 84.09% (95% CI: 72.57-91.46%) and specificity of 88.76% (95% CI: 76.37-95.26%) for ACS. The PPV for ACS was 60% (95% CI: 47.97-71.11%), while the NPV was 100% (95% CI: 90.59-100%). In contrast, the sensitivity for myocarditis was notably lower at 25.81% (95% CI: 4.52-70.70%), with a specificity of 95.12% (95% CI: 86.43-98.69). The PPV for myocarditis was 2.5% (95% CI: 0.45-13.14%), and the NPV was 100% (95% CI: 91.12-100%) (Figure 2) (Table 4). A chi-square test confirmed a statistically significant association between the SMS and the final diagnosis (p<0.001).

**Table 4:** Diagnostic accuracy of SMS ACS: acute coronary syndrome, PPV: positive predictive value, NPV: negative predictive value, SMS: Salzburg Myocarditis Score

Diagnostic metric	ACS	Myocarditis
Sensitivity	84.09%	25.81%
Specificity	88.76%	95.12%
PPV	60%	2.5%
NPV	100%	100%

## Discussion

The SMS is primarily used as a clinical tool to differentiate between ACS and myocarditis in patients presenting with acute chest pain. Our study aimed to evaluate the effectiveness of the SMS in accurately identifying these conditions among a diverse patient population at Saveetha Medical College. The results provide valuable insights into the utility and limitations of the SMS in clinical practice. Our study confirms the findings of previous research regarding the high sensitivity and specificity of the SMS in diagnosing ACS and helps in ruling out probable myocarditis [1]. The score appears to be a valuable tool in ruling out ACS in patients with low scores, potentially reducing the need for unnecessary invasive procedures.

One of the significant strengths of this study is its real-world application of the SMS in a varied patient cohort. By including 100 consecutive adult patients with acute chest pain, the study reflects a broad spectrum of clinical scenarios, enhancing the generalizability of the findings. The use of comprehensive cardiac imaging techniques, such as echocardiography and cardiac MRI, and confirmatory diagnostic procedures, like myocardial biopsy, provides a robust foundation for the accuracy of the diagnoses. This multimodal approach ensures that the diagnoses of ACS and myocarditis are made with high confidence, reducing the likelihood of misclassification.

However, the low sensitivity for myocarditis is a major limitation of the SMS. This finding indicates that a significant proportion of myocarditis cases may be missed if reliance is placed solely on the SMS, aligning with concerns raised about the limitations of relying solely on troponin levels in diagnosing myocarditis [1]. Therefore, it is essential to consider alternative diagnoses and additional diagnostic tests, such as cardiac MRI, when myocarditis is suspected, even in patients with low SMS scores, as recommended by current guidelines [2]. The integration of the SMS with other clinical and diagnostic parameters, such as novel biomarkers or imaging techniques, may enhance its diagnostic utility.

The results of our study confirm that the SMS is highly effective in identifying ACS, with a sensitivity of 84.09% and a specificity of 88.76%. These findings are consistent with previous research suggesting that the SMS can be a valuable tool for ruling out ACS in patients presenting with chest pain. The high NPV of 100% further supports the utility of the SMS in excluding ACS in patients with low scores, potentially reducing the need for unnecessary invasive procedures.

However, the SMS demonstrated a low sensitivity for myocarditis, identifying only one case in this cohort. This finding aligns with concerns raised in previous studies about the limitations of clinical scores in diagnosing myocarditis, particularly given the heterogeneity of the condition and its overlapping symptoms with other cardiac and non-cardiac conditions. The low sensitivity indicates that a significant proportion of myocarditis cases may be missed if reliance is placed solely on the SMS, underscoring the importance of integrating the score with other diagnostic modalities, such as cardiac MRI and biopsy, when myocarditis is suspected.

The study also highlights the prevalence of other conditions presenting with chest pain, such as atypical chest pain and secondary infections. Atypical chest pain, diagnosed in 11 patients, encompasses a range of non-cardiac causes, including gastroesophageal reflux disease, musculoskeletal pain, and anxiety disorders. The identification of secondary infections, such as pneumonia and sepsis, in five patients further illustrates the complexity of diagnosing chest pain in an emergency setting and the need for a thorough clinical evaluation.

In addition to the findings of Mirna et al. [1], studies by Blauwet and Cooper and Cooper [2,3] have emphasized the heterogeneity in the clinical presentation of myocarditis, which complicates its diagnosis based solely on clinical scores. This variability underscores the need for a multifaceted diagnostic approach, combining clinical scores like the SMS with advanced imaging techniques and biomarker profiling. CMR has been highlighted by Friedrich and Marcotte [12] as a particularly effective tool for diagnosing myocarditis, given its ability to detect myocardial inflammation, edema, and fibrosis. CMR is considered the gold standard for non-invasive imaging in myocarditis and can significantly improve diagnostic accuracy when used alongside the SMS.

Furthermore, the pathophysiological mechanisms of myocarditis, as discussed by Imazio and Trinchero [4], indicate that the inflammatory processes can be variable and sometimes patchy, leading to discrepancies in clinical presentation and diagnostic findings. This variability necessitates the use of comprehensive diagnostic protocols that include detailed patient history, clinical examination, ECG changes, cardiac biomarkers, and imaging studies.

The findings of this study support the need for a comprehensive diagnostic approach that integrates clinical scores like the SMS with advanced imaging and biomarker analysis to improve the differentiation between ACS and myocarditis. Future research should focus on refining the SMS by incorporating additional parameters, such as novel biomarkers of myocardial injury and inflammation, to enhance its sensitivity to myocarditis. This could lead to better diagnostic precision and improved patient outcomes in clinical practice.

In the rising trend of ACS and myocarditis. Several scoring systems have been implemented in clinical routines to support diagnostic and therapeutic decisions in patients presenting with chest pain. For example, the Wells score and the Geneva score have been developed to facilitate the diagnosis of suspected pulmonary embolism [10,11], whereas the Aortic Dissection Detection Risk Score has been validated to estimate the pretest probability of aortic dissection [20]. So, the development of new scoring systems is necessary.

Given the limitations identified in this study, future research should aim to address these gaps and build upon the findings presented. Prospective studies with larger, more diverse patient populations would provide a more comprehensive understanding of the utility of the SMS across different clinical settings and patient demographics. Additionally, exploring the integration of advanced imaging techniques, such as FDG PET, and novel biomarkers into the SMS could improve its sensitivity and specificity for myocarditis, enhancing its overall diagnostic utility.

Moreover, comparative studies evaluating the SMS against other clinical scores and diagnostic tools would provide valuable insights into its relative effectiveness and potential applications in various healthcare settings. Finally, research into the pathophysiology and clinical presentation of myocarditis and other atypical causes of chest pain would further inform the development of more accurate and reliable diagnostic algorithms, ultimately improving patient outcomes.

The results suggest that while the SMS is a valuable tool to rule out myocarditis and identify ACS in patients with acute chest pain, its low sensitivity for myocarditis indicates that additional diagnostic tests, such as cardiac MRI, are crucial when myocarditis is suspected. This is particularly important in cases where patients present with ST-segment elevation but are not diagnosed with ACS or myocarditis. In such cases, other causes of ST elevation, such as pericarditis or other inflammatory conditions, should be considered, and further testing may be required to rule out these diagnoses.

## Conclusions

The SMS is a valuable tool to rule out probable myocarditis and helps in the identification of ACS in patients with acute chest pain, offering high sensitivity and specificity for this condition. However, due to its low sensitivity for myocarditis, it should not be relied upon exclusively for diagnosing this condition. A comprehensive diagnostic approach integrating clinical scores with advanced imaging and biomarker analysis is essential for accurate differentiation between ACS and myocarditis. Further studies are warranted to validate the SMS and enhance its diagnostic capabilities, ultimately improving patient outcomes in clinical practice.
